# Pacing in a self-paced world record attempt in 24-h road cycling

**DOI:** 10.1186/s40064-015-1445-1

**Published:** 2015-10-29

**Authors:** Beat Knechtle, Nicola Luigi Bragazzi, Thomas Rosemann, Christoph A. Rüst

**Affiliations:** Facharzt FMH für Allgemeinmedizin, Gesundheitszentrum St. Gallen, Vadianstrasse 26, 9001 St. Gallen, Switzerland; Institute of Primary Care, University of Zurich, Zurich, Switzerland; Department of Health Sciences (DISSAL), School of Public Health, University of Genoa, Genoa, Italy

**Keywords:** Ultra-endurance, Cycling speed, Power output

## Abstract

**Background:**

Pacing strategy plays a major role in sport performance. However, there is a dearth of knowledge concerning pacing during ultra-endurance sport events. The present case study investigated the pacing of an ultra-cyclist in a self-paced attempt to break the world record in 24-h road cycling and, with all the caveats and the limitations affecting a case report, could be useful in generating hypotheses and further studies about pacing dynamics during prolonged sport performances.

**Case description:**

A well experienced ultra-cyclist completed laps of 11.731 km during 24 h and the support crew recorded for each lap time and power output in Watt. The trend in cycling speed and power output across laps was investigated using regression analyses. A mixed-effects regression model including lap, ambient air temperature, air pressure, air humidity and wind speed as fixed variables was used to investigate a relationship of environmental factors with cycling speed.

**Discussion and evaluation:**

The athlete achieved 896.173 km within the 24 h. He set a new world record by breaking the old record (Jure Robic, 2004, 834.77 km) by 61.403 km. He cycled at an average speed of 37.34 km/h with an average power output of 250.2 W. The decrease in cycling speed and power output across laps could be modelled linearly. Temperature and wind speed were related to cycling speed during the whole event. There was a significant interaction air temperature × relative humidity for the whole event.

**Conclusions:**

The athlete adopted a positive pacing (i.e. speed gradually declined throughout the event) and environmental factors (i.e. temperature and wind speed) influenced cycling speed during the event.

## Background

Pacing in athletic performance describes how an athlete controls and distributes work and energy throughout an exercise task (Abbiss and Laursen 2005; 2008). Generally, pacing during athletic performance can be divided in negative (i.e. increase in speed over time), positive (i.e. continual slowing over time), explosive all-out (i.e. maximal speed possible), even (i.e. same speed over time), parabolic-shaped (i.e. positive and negative pacing in different segments of the race, such as U-shaped, J-shaped and reverse J-shaped pacing profiles) and variable (i.e. pacing with multiple fluctuations over time) pacing (Abbiss and Laursen 2008).

Choosing the best pacing approach (‘pacing strategy selection’) is a critical parameter for sport performance, especially in those disciplines in which athletes compete against each other (Thompson 2014). Pacing strategy is complex and multidimensional, since it is the result of different determinants, such as the biological and genetic make-up of the athlete, psychological status, training and competition history, equipment, life experiences, and environment (Mauger 2014; Thompson 2014).

While research has so far focused on pacing strategies during short-duration or extremely short-duration performances, little is known about pacing in ultra-endurance performance (Abbiss and Laursen 2008). It is assumed that athletes in an ultra-endurance event adopt a positive pacing strategy where the athlete progressively slows down after peak speed is reached (Abbiss and Laursen 2008). In their review, Abbiss and Laursen (2008) mentioned positive pacing for triathletes (Laursen et al. 2005) competing in an Ironman and for cyclists competing in the 525-km ‘Race across the Alps’ (Neumayr et al. 2004). For other ultra-endurance athletes such as ultra-runners, recent studies investigated pacing in elite 100-km (Lambert et al. 2004) and 161-km ultra-marathoners (Hoffman 2014; Parise and Hoffman 2011) and recreational 100-km age group ultra-marathoners (Knechtle et al. 2015; Rüst et al. 2015b). Elite 100-km ultra-marathoners ran with fewer changes in speed, started the race at a faster running speed than slower runners, and were able to maintain their initial speed for a longer distance before slowing down compared to slower runners (Lambert et al. 2004). Winners in a 161-km ultra-marathon generally remained relatively close behind the leading runners before taking the lead in the middle half of the race and then avoiding slowing down as much as the other top runners in the latter race stages (Hoffman 2014). Indeed, in a 100-km ultra-marathon, the fastest runners were able to achieve a negative pacing in the last segment of the race (Knechtle et al. 2015). As far as age is concerned, 100-km ultra-marathoners aged 18-24 years were slower than athletes in most other age groups and there was no trend of slowing down for older athletes (Rüst et al. 2015b).

For cyclists, pacing has been mainly investigated in time trials in rather short distances of 250 m (De Jong et al. 2015), 1000 m (Fernandes et al. 2014), and 10,000 m (Barwood et al. 2015). For ultra-distance cycling, we have little knowledge about the pacing strategy in these athletes. Neumayr et al. (2004) investigated changes in heart rate during the ‘Race across the Alps’ and found that exercise intensity declined significantly during the race, as indicated by a decrease in average heart rate/maximum heart rate. In ultra-cyclists competing in the ‘Race Across AMerica’ (RAAM) between 2010 and 2014, positive pacing seemed to be the adequate strategy. The top three finishers started faster and had a higher power output at the start compared to less successful competitors, achieved the highest peak cycling speeds and power output and maintained peak cycling speed and power output longer before slowing down (Heidenfelder et al. 2015).

However, in ultra-triathletes competing in multi-day events (i.e. daily an Ironman triathlon) for 30 days (Knechtle et al. 2014a) and 33 days (Knechtle et al. 2014b), pacing was even where the best athletes were able to maintain their daily Ironman race time for about one month. It was assumed that previous experience was the most important aspect for this even pacing (Knechtle et al. 2014a, b). However, although these triathletes performed ultra-endurance performances, their events were multi-stage races with breaks during the nights and not informative for a non-stop ultra-endurance performance.

Since we have only very little knowledge about the pacing in ultra-distance cycling, the present case study investigated the pacing of one ultra-cyclist in a self-paced attempt to break the world record in 24-h road cycling. This case study is intended to be a pilot, preliminary study. Further research is needed to fill in the gap of knowledge.

## Methods

### Subject

The cyclist (33 years, 76 kg, 1.86 m, BMI 22 kg/m^2^) was an experienced ultra-endurance cyclist with several podiums in ultra-cycling races such as the ‘RAAM’, the ‘Race around Slovenia’, the ‘Race across Italy’ and the ‘Race around Ireland’. Overall, he had completed 11 24-h cycling races with a personal record of 950 km in a draft-legal 24 h cycling race (http://www.christophstrasser.at/erfolge_berichte_archiv/erfolge/). Informed consent was obtained from the athlete and he agreed to the analysis and publication of his data as presented in this article.

### The event

On March 20th 2015, the athlete started his self-pace record attempt at the airport Berlin-Tempelhof, in Berlin, Germany. The athlete used the runway of the airport and performed laps of 11.731 km around the runway of the airport. One lap consisted of an ‘8’ with a start and finish area. He used a conventional time trial bike (S-WORKS, Specialized) with a disc wheel. His nutrition consisted mainly of Ensure^®^. From time to time, he ingested magnesium and caffeine during the night. In total, he made 10 breaks to go to the toilet and for changes of clothes and the bike. At 01:00 p.m. of the second day, he had a flat tire. He used a special dress for time trials (http://www.owayo.ch/radsport-zeitfahranzuege.htm) during the cold in the night (Table 1).

**Table 1:** Temperature, barometric pressure, humidity, wind direction and wind speed during the 24 h (W = west, S = south, N = north)

Time	Temperature (°C)	Barometric pressure (hPa)	Relative humidity (%)	Wind direction	Wind speed (m/s)
03:00 p.m.	13.1	762.1	28	W	3
04:00 p.m.	13.1	761.5	28	SWSW	4
05:00 p.m.	12.4	760.9	30	W	3
06:00 p.m.	11.4	760.6	33	W	3
07:00 p.m.	10.6	760.2	35	WSW	2
08:00 p.m.	9.5	760.0	39	SW	2
09:00 p.m.	8.8	759.8	41	SW	2
10:00 p.m.	7.6	759.4	46	SW	2
11:00 p.m.	6.7	759.0	49	SW	3
12:00 p.m.	5.6	758.5	55	WSW	2
01:00 a.m.	4.5	757.9	63	SW	2
02:00 a.m.	2.8	757.9	74	WNW	3
03:00 a.m.	2	757.6	83	WNW	3
04:00 a.m.	2.6	757.1	86	S	2
05:00 a.m.	2.1	756.9	88	S	2
06:00 a.m.	2.1	756.7	91	S	3
07:00 a.m.	2.2	756.4	89	WSW	2
08:00 a.m.	2.5	756.2	88	SSW	3
09:00 a.m.	3.6	756.3	83	SW	4
10:00 a.m.	5.1	756.3	78	SWSW	3
11:00 a.m.	5.3	756.4	78	SWSW	4
12:00 0.m.	5.4	756.4	78	S	3
01:00 p.m.	5	756.4	81	S	4
02:00 p.m.	5.1	756.5	81	W	3
03:00 p.m.	5.1	756.5	81	WNW	3

### Methods

Lap times, cycling speed (km/h) for each lap (distance/time for each lap) and power output in Watt (W) for each lap were recorded by the support crew of the athlete and we obtained the data from the website of the athlete (http://www.christophstrasser.at/24h_road_rekord_2015/). Power output in W was measured while cycling using power2max TYPE S (http://www.power2max.de/europe/power2max-type-s/). Weather data were obtained from the local weather station from the airport Berlin Tempelhof (http://rp5.md/Wetterarchiv_in_Berlin,_Tempelhof) (Table 1).

### Statistical analysis

Case-studies and case series, by definition, cannot be used in order to establish cause-effect relationship, both because of the limited, underpowered sample size and the intrinsic nature of a case report, whose findings cannot be generalizable to the entire population and should be interpreted with caveat. For this reason, the usage of inferential statistics is usually considered wrong and descriptive statistics is preferred. However, there are some exceptions to this rule. For example, in case of longitudinal studies, when data are recorded over time, even for a single subject, inferential approaches can result fruitful, when comparing or controlling for multiple variables and/or analysing time trend. On the other hand, due to the shortcomings that characterize a single case-report, caution should be taken into interpreting the results and further research in the field is mandatory for obtaining findings, not contaminated by spurious linkages (Encyclopaedia of case study research, 2009). Each set of data was tested for normal distribution (D’Agostino and Pearson omnibus normality test) before statistical analyses. To investigate trends in cycling speed and power output across laps, a test for linear trend was performed and the best fitting linear regression model was computed. Correlations between distance, cycling speed and power were estimated. Pearson correlation was used in case of normal distributed data and Spearman correlation was used in case of not normally distributed data. Additionally, a test for linear trend was performed. To investigate changes in cycling speed and power output during the event, a mixed-effects regression model was used for the whole event. We included the number of laps, ambient air temperature, air pressure, air humidity and wind speed as fixed variables. We also considered interaction effects between the meteorological parameters. We decided to include these parameters because it seems that external factors (such as environmental parameters) play an important role in pacing dynamics during particularly prolonged sport events (Abbiss and Laursen 2008), such as the performance object of the current study. Statistical analyses were performed using CurveExpert Professional (Version 2.0.3, Hyams D.G.) and GraphPad Prism (Version 6.01, GraphPad Software, La Jolla, CA, USA). Significance was accepted at *p* < 0.05 (two-sided for *t*-tests). Data in the text and figures are given as mean ± standard deviation (SD).

## Results

The athlete completed a total of 76 full laps of 11.731 km plus 4.6 km and achieved 896.173 km during the 24 h. He set a new world record in 24-h road cycling by breaking the old record of 834.77 km held by Jure Robic since 2004 by 61.403 km. On average, he was riding at a mean cycling speed of 37.34 km/h and achieved an average power output of 250.2 W.

**Fig. 1:**
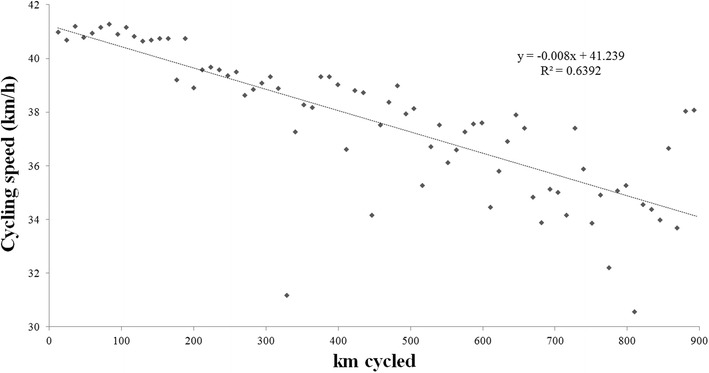
Changes in cycling speed (km/h) across laps

**Fig. 2:**
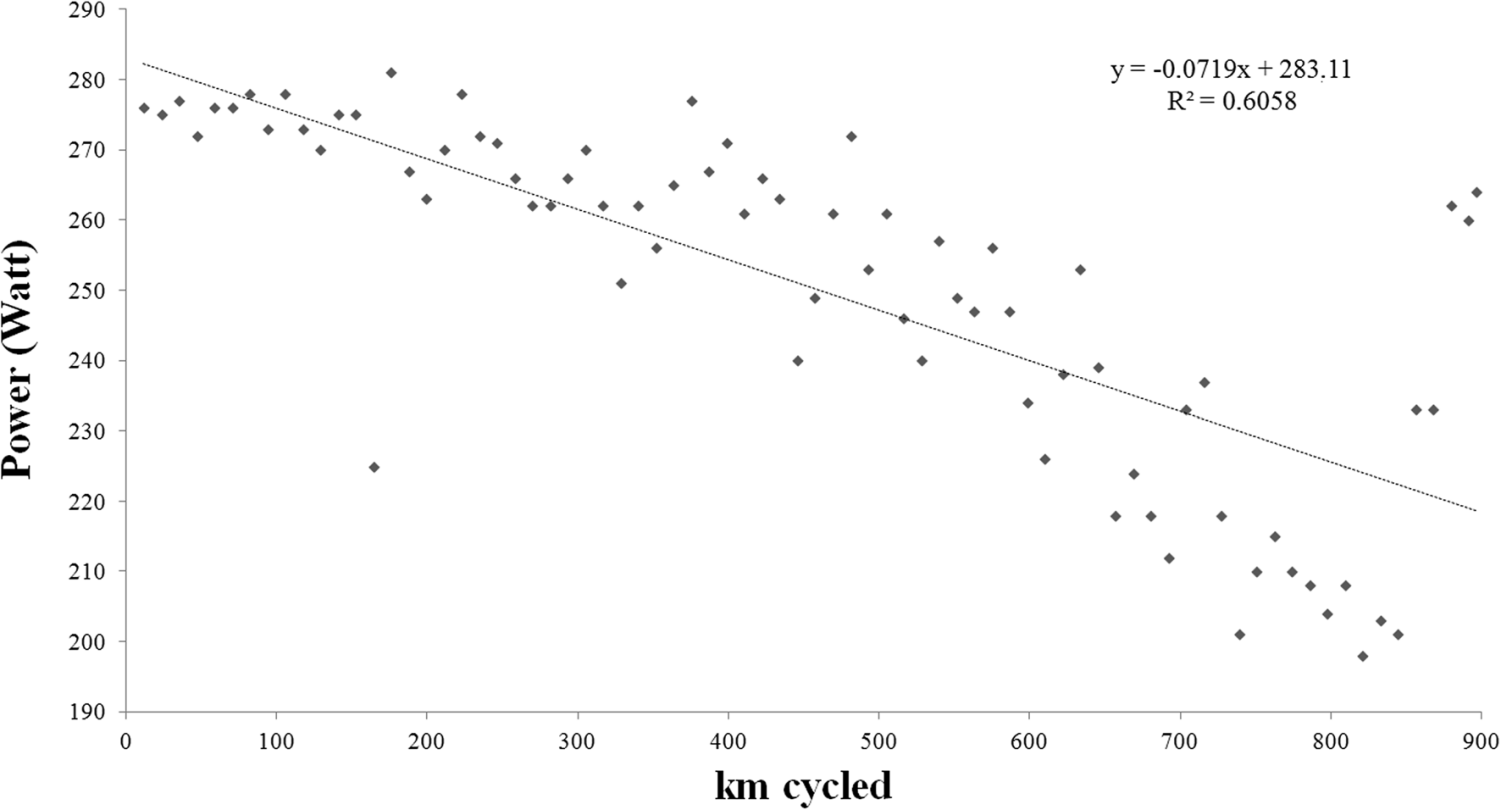
Changes in power output (W) across laps

Data for cycling speed were normally distributed; data for power output were, however, not normally distributed. The achieved distance correlated significantly and negatively to cycling speed (r = −0.79, *p* < 0.0001) (Fig. 1) and power output (r = −0.85, *p* < 0.0001) (Fig. 2).

Ambient air temperature and wind speed were related to cycling speed when the whole event was considered (Table 2). There was a significant interaction air temperature × relative humidity for the whole event.

**Table 2:** Results of the mixed-effects regression analyses for the whole event regarding the relationship of temperature, pressure, humidity and wind speed with cycling speed

Parameter	Estimate	*P* value
Temperature	−0.274	0.040*
Barometric pressure	−0.151	0.665
Relative humidity	−0.001	0.952
Wind speed	0.373	0.017*
Temperature × humidity	0.007	0.032*

* Significant *p*-values

## Discussion

We investigated the changes in cycling speed and power output across laps and across segments in an ultra-cyclist in a self-paced attempting to break the world record in 24-hour road cycling. The main findings were (1) the decrease in cycling speed and power output across laps could be modelled linearly and, (2) ambient air temperature and wind speed were related to cycling speed for the whole event.

### Positive pacing during 24 h

During the 24 h, cycling speed and power output decreased linearly across laps leading to a positive pacing. This finding is similar to the pacing in ultra-cyclists competing in the ‘RAAM’ between 2010 and 2014 where a positive pacing seemed to be the best strategy (Heidenfelder et al. 2015). Unfortunately, we are not able to compare and quantify the decrease in performance between a 24-h cycling event and the ‘RAAM’ due to the different length and duration of the two events.

The continuous decrease in performance was most probably due to several different factors such as a high energy deficiency (Bescós et al. 2012b; Knechtle et al. 2005; Stewart and Stewart 2007), an increase in energy cost (Brisswalter et al. 1998), a continuous depletion of triglyceride and glycogen in skeletal muscles (Enqvist et al. 2010; Johnson et al. 2004; Stellingwerff et al. 2007) and a shift from glycogen to fat as the major energy source (Farley and Hamley 1978; Knechtle et al. 2003). For example, an official finisher in a 24-h ultra-cycling race suffered an energy deficit of ~9915 kcal (Bescós et al. 2012b). Another case report investigating one ultra-endurance athlete during the attempt on the record for the longest period of stationary cycling showed an energy deficit of ~3290 kcal while covering 1126 km and cycling at a mean speed of 24 ± 1.6 km/h for 46.7 h (Stewart and Stewart 2007).

Exercise duration appears to have the most significant influence on both the self-selected and the optimal pacing strategies selected by athletes during a competition (Abbiss and Laursen 2008). The most successful cyclists are known to have a high maximal aerobic power output and an ability to work at relatively high power outputs for long periods (Atkinson et al. 2003). However, the regulation of pacing seems to be a complex process influenced by many intrinsic and extrinsic factors (Wu et al. 2014). Ultra-endurance athletes routinely encounter a number of physiological and environmental challenges such as an insufficient energy intake (Hulton et al. 2010; Knechtle et al. 2005), diet-related problems such as nausea, feeling of fullness, and abdominal distension (Lindeman 1991; Stuempfle and Hoffman 2015) and dehydration (Bowen et al. 2006; Moyen et al. 2015). Other problems such as sleep deprivation (Knechtle et al. 2005, 2012; Lahart et al. 2013; Smith et al. 1998) and intense unwanted emotions (Lane and Wilson 2011; Pedlar et al. 2007) might also occur where all might negatively influence ultra-endurance performance.

After 22:37 h:min, the athlete had already broken the old world record. Maybe he was motivated to attack and break the limit of 900 km which might explain the fact that he increased cycling speed in the last stage of the event. In other terms, the improvement of 61.403 km was performed within 1:23 h:min at an average cycling speed of 44.38 km/h. This speed was considerably faster than the mean cycling speed of 37.34 km/h for the whole event. One might assume that the last laps influenced the analysis of the trend. We compared the results with and without the last laps where cycling speed increased. However, the results were not different regarding the non-linear decrease in cycling speed and power output.

The near finish or the possibility for a new record most probably motivated the athlete. In a recent case study describing a swimmer crossing the Adriatic Sea in a 78.1-km solo ultra-endurance open-water swim within 23:44 h:min, the athlete increased swimming speed and stroke length between 21 h and the end (De Ioannon et al. 2014). Decreases in both swimming speed and in stroke length were observed between 18 h and 21 h compared to the first 3 h of the event. The authors assumed that the observed increases in swimming speed at the end of the event were due to the high motivation of the swimmer to accomplish this unique challenge (De Ioannon et al. 2014). In a recent study investigating the performance in 100-km ultra-marathoners across segments, the best pacers were able to achieve a negative pacing in the last segment. The authors speculated that environmental and motivational reasons (i.e. early dawn, flat circuit) were the most likely reasons for a negative pacing (Knechtle et al. 2015).

### Influence of environmental conditions

An interesting finding was that the athlete cycled at a mean air temperature of ~6 °C where the lowest temperature was ~2 °C at 03:00 a.m. Air temperature and wind speed were related to cycling speed during the whole event. Wind speed has indeed a complex impact on performance, for example, it might, have an influence on a hilly course. In a case study, the performance for a 70-kg cyclist on a 10-km time trial with alternating 1-km segments of uphill and downhill was modelled. The model predicted that significantly time savings could be realized on a hilly and windy course by slightly increasing power on uphill or headwind segments while compensating with reduced power on downhill or tailwind segments (Swain 1997). However, this was not taken into account and modelled in our case study.

In the ‘Furnace Creek 508’ where ultra-cyclists compete in Death Valley at temperatures up to 30–40 °C, cycling speed was not influenced by air temperature (Rüst et al. 2015a). The fastest male finishers compete at 27.1 ± 0.7 km/h. In the ‘RAAM’, the change in temperature and altitude had a significant and positive influence on cycling speed in all finishers, but not in age group 50–59 years. Temperature had a positive and altitude a negative influence on power output in all finishers (Heidenfelder et al. 2015). When power output was estimated using wind speed, also power output decreased across time stations similarly to cycling speed.

Similarly to the present case report, rather low cycling speeds seem not to be influenced by ambient temperatures. In ultra-marathoners, however, ambient temperatures seem to have an influence on performance (Parise and Hoffman 2011). In a 161-km ultra-marathon, extreme heat impaired all runners’ ability where faster runners were at a greater disadvantage (Parise and Hoffman 2011). Also for marathoners, air temperature and performance are significantly correlated. When the temperature increases above an optimal level, running speed decreases (El Helou et al. 2012). In cycling, however, hyperthermia (Nybo 2010) might impair performance rather than a cold environment (Crampton et al. 2013).

### Limitations

Our analysis has some limitations, besides those affecting a case study and that already mentioned of not having modelled the course according to uphill and downhill segments, best capturing the real pacing dynamics. Furthermore, this analysis is limited due to the fact that some important parameters influencing the pacing strategy (Thompson 2014) were not analysed, such as the biological make-up of the athlete or training and performance history. Moreover, the aspect of food and fluid intake was not considered (Bescós et al. 2012a, b; Chlíbková et al. 2014). Appropriate nutrition during ultra-cycling has been shown as a key variable (Knechtle et al. 2011b) and dehydration may adversely affect mood state and perceptual ratings during ultra-endurance cycling (Moyen et al. 2015). A further limitation is that we have no data of exercise intensity (i.e. heart rate or percent of maximum oxygen uptake during the performance) (Knechtle et al. 2011a). With records of heart rate, carbohydrate and fat oxidation might have been estimated. In a case report of a 24-h cyclist, the percentage of fat oxidation increased from 47 to 57 % and the percentage of carbohydrate oxidation decreased from 52 to 42 % (Knechtle et al. 2003). The athlete made in total 10 breaks (i.e. toilet, changes of clothes, flat tire) during the 24 h. The inclusion of laps with interruption in the analysis (i.e. outliers) might impact the line of best fit. We used data from the power meter power2max TYPE S. However, no study exists on the reliability of this device. A further limitations is that the authors were not involved in the data collection, that data posted on the internet from the participant was used in all data analysis.

## Conclusions

To summarize, the athlete set a new world record in 24-h road cycling in his self-paced attempt and adopted a positive pacing (i.e. gradual decline of cycling speed throughout the duration of the event). Environmental factors (i.e. temperature and wind speed) correlated with the performance. Although he cycled at a mean temperature of ~6 °C, he was able to break the world record.

## References

[CR1] Abbiss CR, Laursen PB (2005). Models to explain fatigue during prolonged endurance cycling. Sports Med.

[CR2] Abbiss CR, Laursen PB (2008). Describing and understanding pacing strategies during athletic competition. Sports Med.

[CR3] Atkinson G, Davison R, Jeukendrup A, Passfield L (2003). Science and cycling: current knowledge and future directions for research. J Sports Sci.

[CR4] Barwood MJ, Corbett J, Wagstaff CR, McVeigh D, Thelwell RC (2015). Improvement of 10-km time-trial cycling with motivational self-talk compared with neutral self-talk. Int J Sports Physiol Perform.

[CR5] Bescós R, Rodríguez FA, Iglesias X, Knechtle B, Benítez A, Marina M, Padullés JM, Torrado P, Vazquez J, Rosemann T (2012). Nutritional behavior of cyclists during a 24-hour team relay race: a field study report. J Int Soc Sports Nutr.

[CR6] Bescós R, Rodríguez FA, Iglesias X, Benítez A, Marina M, Padullés JM, Torrado P, Vázquez J, Knechtle B (2012). High energy deficit in an ultraendurance athlete in a 24-hour ultracycling race. Proc (Bayl Univ Med Cent).

[CR7] Bowen RL, Adams JH, Myburgh KH (2006). Nausea and high serum osmolality during a simulated ultra-endurance adventure race: a case-control study. Int J Sports Physiol Perform.

[CR8] Brisswalter J, Fougeron B, Legros P (1998). Variability in energy cost and walking gait during race walking in competitive race walkers. Med Sci Sports Exerc.

[CR9] Chlíbková D, Knechtle B, Rosemann T, Tomášková I, Chadim V, Shortall M (2014). Nutrition habits in 24-hour mountain bike racers. Springerplus.

[CR10] Crampton D, Donne B, Warmington SA, Egaña M (2013). Cycling time to failure is better maintained by cold than contrast or thermoneutral lower-body water immersion in normothermia. Eur J Appl Physiol.

[CR11] De Ioannon G, Cibelli G, Mignardi S, Antonelli A, Capranica L, Piacentini MF (2014) Pacing and mood changes while crossing the Adriatic Sea from Italy to Albania: a case study. Int J Sports Physiol Perform [Epub ahead of print] 10.1123/ijspp.2014-0264 25310382

[CR12] De Jong J, van der Meijden L, Hamby S, Suckow S, Dodge C, de Koning JJ, Foster C (2015) Pacing strategy in short cycling time trials. Int J Sports Physiol Perform. 2015. [Epub ahead of print] 10.1123/ijspp.2014-009 25756313

[CR13] El Helou N, Tafflet M, Berthelot G, Tolaini J, Marc A, Guillaume M, Hausswirth C, Toussaint JF (2012). Impact of environmental parameters on marathon running performance. PLoS One.

[CR14] Encyclopedia of Case Study Research (2009). In: Albert J. Mills, Gabrielle Durepos, Elden Wiebe (eds). SAGE Publications, Inc, p 152. ISBN-10: 1412956706; ISBN-13: 978-1412956703

[CR15] Enqvist JK, Mattsson CM, Johansson PH, Brink-Elfegoun T, Bakkman L, Ekblom BT (2010). Energy turnover during 24 hours and 6 days of adventure racing. J Sports Sci.

[CR16] Farley GR, Hamley EJ (1978). Progressive changes in energy cost during a three hour race walk exercise. Br J Sports Med.

[CR17] Fernandes AL, Lopes-Silva JP, Bertuzzi R, Casarini DE, Arita DY, Bishop DJ, Lima-Silva AE (2014). Effect of time of day on performance, hormonal and metabolic response during a 1000-M cycling time trial. PLoS One.

[CR18] Heidenfelder A, Rosemann T, Rüst CA, Knechtle B (2015) Pacing strategies of ultra-cyclists at the ‘Race Across AMerica’. Int J Sports Physiol Perform 2015. [Epub ahead of print] 10.1123/ijspp.2015-0051 26215121

[CR19] Hoffman MD (2014). Pacing by winners of a 161-km mountain ultramarathon. Int J Sports Physiol Perform.

[CR20] Hulton AT, Lahart I, Williams KL, Godfrey R, Charlesworth S, Wilson M, Pedlar C, Whyte G (2010). Energy expenditure in the Race Across America (RAAM). Int J Sports Med.

[CR21] Johnson NA, Stannard SR, Thompson MW (2004). Muscle triglyceride and glycogen in endurance exercise: implications for performance. Sports Med.

[CR22] Knechtle B, Knechtle P, Müller G, Zwyssig D (2003). Energieumsatz an einem 24 Stunden Radrennen: Verhalten von Körpergewicht und Subkutanfett. Österreichisches Journal für Sportmedizin.

[CR23] Knechtle B, Enggist A, Jehle T (2005). Energy turnover at the Race Across America (RAAM): a case report. Int J Sports Med.

[CR24] Knechtle B, Knechtle P, Kohler G (2011). The effect of 1000 km nonstop cycling on fat mass and skeletal muscle mass. Res Sports Med.

[CR25] Knechtle B, Knechtle P, Rüst CA, Rosemann T, Lepers R (2011). Finishers and nonfinishers in the ‘Swiss Cycling Marathon ‘ to qualify for the ‘Race Across America ‘. J Strength Cond Res.

[CR26] Knechtle B, Wirth A, Knechtle P, Rüst CA, Rosemann T, Lepers R (2012). No improvement in race performance by naps in male ultra-endurance cyclists in a 600-km ultra-cycling race. Chin J Physiol.

[CR27] Knechtle B, Rosemann T, Lepers R, Rüst CA (2014). A comparison of performance of Deca Iron and Triple Deca Iron ultra-triathletes. Springerplus.

[CR28] Knechtle B, Rüst CA, Rosemann T, Martin N (2014). 33 Ironman triathlons in 33 days-a case study. Springerplus.

[CR29] Knechtle B, Rosemann T, Zingg MA, Stiefel M, Rüst CA (2015). Pacing strategy in male elite and age group 100-km ultra-marathoners. Open Access J Sports Med.

[CR30] Lahart IM, Lane AM, Hulton A, Williams K, Godfrey R, Pedlar C, Wilson MG, Whyte GP (2013). Challenges in maintaining emotion regulation in a sleep and energy deprived state induced by the 4800 Km ultra-endurance bicycle race; The Race Across AMerica (RAAM). J Sports Sci Med.

[CR31] Lambert MI, Dugas JP, Kirkman MC, Mokone GG, Waldeck MR (2004). Changes in running speeds in a 100 km ultra-marathon Race. J Sports Sci Med.

[CR32] Lane AM, Wilson (2011). Emotions and emotional intelligence among ultra-endurance runners. J Sci Med Sports.

[CR33] Laursen PB, Knez WL, Shing CM, Langill RH, Rhodes EC, Jenkins DG (2005). Relationship between laboratory-measured variables and heart rate during an ultra-endurance triathlon. J Sports Sci.

[CR34] Lindeman AK (1991). Nutrient intake of an ultra-endurance cyclist. Int J Sport Nutr.

[CR35] Mauger AR (2014). Factors affecting the regulation of pacing: current perspectives. Open Access J Sports Med.

[CR36] Moyen NE, Ganio MS, Wiersma LD, Kavouras SA, Gray M, McDermott BP, Adams JD, Binns AP, Judelson DA, McKenzie AL, Johnson EC, Muñoz CX, Kunces LJ, Armstrong LE (2015). Hydration status affects mood state and pain sensation during ultra-endurance cycling. J Sports Sci.

[CR37] Neumayr G, Pfister R, Mitterbauer G, Maurer A, Hoertnagl H (2004). Effect of ultramarathon cycling on the heart rate in elite cyclists. Br J Sports Med.

[CR38] Nybo L (2010). Cycling in the heat: performance perspectives and cerebral challenges. Scand J Med Sci Sports.

[CR39] Parise CA, Hoffman MD (2011). Influence of temperature and performance level on pacing a 161 km trail ultramarathon. Int J Sports Physiol Perform.

[CR40] Pedlar CR, Lane AM, Lloyd JC, Dawson J, Emegbo S, Stanley N (2007). Sleep profiles and mood states during an expedition to the South Pole. Wilderness Environ Med.

[CR41] Rüst CA, Rosemann T, Lepers R, Knechtle B (2015). Gender difference in cycling speed and age of winning performers in ultra-cycling—the 508-mile “Furnace Creek” from 1983 to 2012. J Sports Sci.

[CR42] Rüst CA, Rosemann T, Zingg MA, Knechtle B (2015). Do non-elite older runners slow down more than younger runners in a 100 km ultra-marathon?. BMC Sports Sci Med Rehabil.

[CR43] Smith RS, Walsh J, Dement W (1998). Sleep deprivation and the race across America. J Sleep.

[CR44] Stellingwerff T, Boon H, Jonkers RA, Senden JM, Spriet LL, Koopman R, van Loon LJ (2007). Significant intramyocellular lipid use during prolonged cycling in endurance-trained males as assessed by three different methodologies. Am J Physiol Endocrinol Metab.

[CR45] Stewart IB, Stewart KL (2007). Energy balance during two days of continuous stationary cycling. J Int Soc Sports Nutr.

[CR46] Stuempfle KJ, Hoffman MD (2015). Gastrointestinal distress is common during a 161-km ultramarathon. J Sports Sci.

[CR47] Swain DP (1997). A model for optimizing cycling performance by varying power on hills and in wind. Med Sci Sports Exerc.

[CR48] Thompson K (2014) Pacing: Individual Strategies for Optimal Performance. Human Kinetics, Champaign. pp 1–240

[CR49] Wu SS, Peiffer JJ, Brisswalter J, Nosaka K, Abbiss CR (2014). Factors influencing pacing in Triathlon. Open Access J Sports Med.

